# Complications of hyperglycaemia with PI3K–AKT–mTOR inhibitors in patients with advanced solid tumours on Phase I clinical trials

**DOI:** 10.1038/bjc.2015.373

**Published:** 2015-11-10

**Authors:** E Geuna, D Roda, S Rafii, B Jimenez, M Capelan, K Rihawi, F Montemurro, T A Yap, S B Kaye, J S De Bono, L R Molife, U Banerji

**Affiliations:** 1 Drug Development Unit, The Institute of Cancer Research and the Royal Marsden NHS Foundation Trust, London, UK; 2 Investigative Clinical Oncology (INCO), Fondazione del Piemonte per l'Oncologia-Institute for Cancer Research and Treatment of Candiolo, Turin, Italy

**Keywords:** Hyperglycaemia, PI3K–AKT–mTOR pathway, PI3K inhibitors, AKT inhibitors, mTOR inhibitors, metabolic complications

## Abstract

**Background:**

PI3K–AKT–mTOR inhibitors (PAMi) are promising anticancer treatments. Hyperglycaemia is a mechanism-based toxicity of these agents and is becoming increasingly important with their use in larger numbers of patients.

**Methods:**

Retrospective case-control study comparing incidence and severity of hyperglycaemia (all grades) between a case group of 387 patients treated on 18 phase I clinical trials with PAMi (78 patients with PI3Ki, 138 with mTORi, 144 with AKTi and 27 with PI3K/mTORi) and a control group of 109 patients treated on 10 phase I clinical trials with agents not directly targeting the PAM pathway. Diabetic patients were excluded in both groups.

**Results:**

The incidence of hyperglycaemia was not significantly different between cases and controls (86.6% *vs* 80.7%, respectively, *P*=0.129). However, high grade (grade 3–4) hyperglycaemia was more frequent in the PAMi group than in controls (6.7% *vs* 0%, respectively, *P*=0.005). The incidence of grade 3–4 hyperglycaemia was greater with AKT and multikinase inhibitors compared with other PAMi (*P*<0.001). All patients with high-grade hyperglycaemia received antihyperglycemic treatment and none developed severe metabolic complications (diabetic ketoacidosis or hyperosmolar hyperglycemic nonketotic state). High-grade hyperglycaemia was the cause of permanent PAMi discontinuation in nine patients.

**Conclusions:**

PI3K–AKT–mTOR inhibitors are associated with small (6.7%) but statistically significant increased risk of high-grade hyperglycaemia compared with non-PAM targeting agents. However, PAMi-induced hyperglycaemia was not found to be associated with severe metabolic complications in this non-diabetic population of patients with advanced cancers.

The PI3K–AKT–mTOR (PAM) signalling pathway is involved in essential cellular functions, including cell proliferation, differentiation, metabolism, survival and angiogenesis (Schmelzle and Hall, 2000; Engelman, 2009). Mutations of the catalytic isoform p110*α* gene-encoding PI3K (*PIK3CA*) and loss of function of the tumour suppressor gene phosphatase and tensin homologue (*PTEN*), a PI3K signalling inhibitor, lead to activation of this pathway and are frequently observed in human cancers (Song *et al*, 2012). This provided the rationale for the development of multiple molecular targeted agents targeting key kinases, PI3K, AKT and mammalian targets of rapamycin (mTOR) along the PAM signal transduction pathway. mTOR inhibitors (mTORi) have been approved for the treatment of advanced renal cell cancer (Hudes *et al*, 2007; Motzer *et al*, 2008), postmenopausal hormone receptor-positive breast cancer (Baselga *et al*, 2012), pancreatic neuroendocrine cancer (Yao *et al*, 2011) and sub-ependymal giant cell astrocytomas (Franz *et al*, 2013). The licensing of mTORi clearly demonstrates the therapeutic potential of targeting the PAM pathway for cancer treatment.

Inhibition of this pathway also affects normal cellular functions, including proliferation, angiogenesis, apoptosis and metabolism. One crucial function of PAM pathway in normal cells is the regulation of tissue metabolism and glucose homeostasis. PI3K, AKT and mTOR are considered essential for the insulin signalling pathway, regulating glucose uptake and glycogen synthesis (Saltiel and Kahn, 2001). PI3K activity and in particular the p110*α* subunit is critical in glucose homeostasis (Engelman *et al*, 2006; Foukas *et al*, 2006; Luo *et al*, 2006). Animal studies have also demonstrated that p110*α* inhibitors, but not p110*β* or p110*δ*, block adipocyte insulin-dependent glucose uptake and regulation (Falasca *et al*, 2007). It has also been shown that AKT regulates hepatic glycogenolysis and glucose uptake through its substrate glycogen synthase kinase-3 (GSK-3; Crouthamel *et al*, 2009). In addition, mTOR is important for insulin production and secretion in response to nutrients. Mammalian targets of rapamycin also promotes beta-cell growth and proliferation in the pancreas and subsequently increases insulin secretion and reduces blood glucose levels (Khamzina *et al*, 2005; Laplante and Sabatini, 2012). In particular, mTORC1 has a crucial role in end organ insulin uptake and its chronic activation, as shown in preclinical models, is associated with insulin resistance through ribosomal protein S6 kinase beta-1 (S6K1; Um *et al*, 2004; Khamzina *et al*, 2005; Laplante and Sabatini, 2012). Finally, liver mTORC2 is essential for insulin-induced AKT signalling regulation and its deletion or inactivation leads to dysregulated hepatic gluconeogenesis and glycogenolysis (Hagiwara *et al*, 2012). Taken together, the above mechanisms provide a comprehensive explanation for the observation that inhibition of PI3K–AKT–mTOR pathway can lead to abrogated insulin function, impaired insulin secretion and development of insulin resistance (Crouthamel *et al*, 2009; Houde *et al*, 2010; Lamming *et al*, 2012; Fang *et al*, 2013).

Hyperglycaemia is well established as an important mechanism-based toxicity associated with mTORi, which has led to development of guidelines for its effective management (Busaidy *et al*, 2012). In patients treated with everolimus and temsirolimus in large phase III clinical trials, incidence of all-grade hyperglycaemia ranged between 12 and 50% with 4–22% of high-grade (G3–G4; Hudes *et al*, 2007; Motzer *et al*, 2008; Verges *et al*, 2014). A recent meta-analysis studying toxicities of allosteric mTORi showed an increased risk of hyperglycaemia, all-grade by 2.95-fold (95% CI, 2.14, 4.05) and high-grade by 5.25-fold (95% CI, 3.07, 9.00) (Sivendran *et al*, 2014).

Although an increased risk of hyperglycaemia has also been described individually for inhibitors of PI3K, AKT and mTOR in phase I–II clinical trials, the severity and complications of hyperglycaemia caused by PAM pathway inhibitors as a whole, together with its appropriate management, has not been studied together. To address this, we have undertaken a comprehensive retrospective case-control analysis to compare incidence and severity of hyperglycaemia across a range of PAMi phase I trials in our center.

## Patients and methods

This is a retrospective case-control study of 496 patients with advanced solid cancer treated on 28 different phase I clinical trials at the Drug Development Unit at The Royal Marsden Hospital (RMH) and Institute of Cancer Research, London, UK between June 2008 and May 2013. The list of trials for cases and controls is summarised in Table 1. Study subjects in the cases group consisted of 387 patients treated on 18 phase I trials involving PI3K, AKT, mTOR and multikinase PI3K/mTOR inhibitors, both as single agents or in combination with cytotoxic chemotherapy or other targeted agents. These trials included four trials with PI3Ki (2 pan PI3K inhibitors, 1 PIK3*α* subunit and 1 PIK3*β* subunit-specific inhibitor), eight trials with AKTi, five trials with dual mTORC 1/2 inhibitors and one multikinase PI3K-mTORC 1/2 inhibitor. Also, 29 patients were treated with combination of PAMi and either cytotoxic chemotherapy or other targeted agents not known to increase the risk of hyperglycaemia such as EGFR and MEK inhibitors. In particular, two of the 18 trials (13 patients) included combinations with platinum or taxane compounds, which included standard steroid premedication.

Furthermore, 109 patients treated on 10 single-agent targeted therapy phase I trials not directly targeting PAM pathway were selected as controls. To reduce the risk of selection bias for the controls, we randomly selected these 10 trials via a sealed envelope method and, subsequently, 109 patients randomly selected using a random number table. Trials included in the control group predominantly targeted growth factor receptors, DNA repair and chromatin remodelling, angiogenesis or miscellaneous targets, without major interactions with PAM pathway.

The control sample size (*n*=109) was calculated applying a bilateral statistical analysis with type I error probability equal to 0.05, power equal to 80% and with an assumption of predicted odds ratio of 4 for grade 3–4 hyperglycaemia in less than 5% of patients in the control group.

We analysed fasting blood glucose level at baseline and the highest blood glucose (fasting or non-fasting) on study to enable us to capture hyperglycaemia at different time points of the day. We analysed the incidence of all-grade and high-grade hyperglycaemia, defined as per NCI CTCAE v 4.0, with a blood glucose level of more than 13.9 mmol l^−1^, requiring treatment according to trial protocols and hospital guidelines.

We only included patients on PAMi trials for which the study results either had been published or presented in scientific meetings. The project proposal was reviewed and approved by the Institutional Audit Committee (DDU056).

As well as patients' demographics, tumour characteristics and treatment data, baseline and highest blood glucose levels during trial were collected. In addition, we collected data on drug interruptions or dose reductions due to hyperglycaemia. Baseline characteristics were well balanced between both study groups as shown in Table 2. Furthermore, no diabetic patients have been included for the analysis both in cases and controls.

All data were extracted from hospital electronic patients' records, anonymised, entered into an Access database (Microsoft, USA) and exported into SPSS (IBM Corp., Version 19.0) for data analysis. Categorical variables were compared by the χ^2^-test. For continuous variables, mean values were compared by Student's test. Where needed, data were log-transformed before statistical comparisons. Correlation between glucose and not normally distributed variables as glycosylated haemoglobin (HbA1c), insulin and c-peptide, was studied with Spearman's test (Supplementary Figures 1–3). The probability to develop high-grade hyperglycaemia according to PAMi type was studied by multivariable binary logistic regression analysis, correcting for other relevant potential explanatory covariates in our data set. Proportions and point estimates are reported together with 95% confidence intervals (CI).

## Results

### Patient characteristics

This study patient population reflects typical phase I patients as previously described from our institution (Molife *et al*, 2012). Median age at time of recruitment to trial was 59.7 years for the cases and 56.3 years for controls. Other baseline characteristics such as age, sex, BMI, tumour type, performance status (PS), RMH prognostic score (Arkenau *et al*, 2008) were also balanced between cases and controls. Most of the patients had a PS of 1 or 2 with a RMH score of 0 to 1. Most common tumour types were colorectal, gynaecological and lung malignancies. Of note, cases comprised more gynaecological tumours compared with controls, however, this has not any effect on the risk of developing hyperglycaemia.

Importantly, inclusion criteria for PAMi trials exclude diabetic patients. For this reason, only non-diabetic patients were entered into random selection method for the control group. Median duration of time on trials was 94.6 (range 1–1096) days for the cases, and 124.7 (range 1–1096) days for the controls.

### Glucose and related blood tests

In addition to glucose, data was available for HbA1c, insulin and c-peptide for 340, 336 and 318 patients treated with PAM inhibitors, respectively. As expected, there was a correlation between glucose and HbA1c, insulin and c-peptide; *P*=0.005, *P*<0.0001 and *P*<0.0001, respectively (Supplementary Figures 1–3). We did not have data for these parameters for the control group of trials of non-PAM inhibitors thus comparison was not possible.

### Increased risk of hyperglycaemia with PAM inhibitors

**Figure 1:**
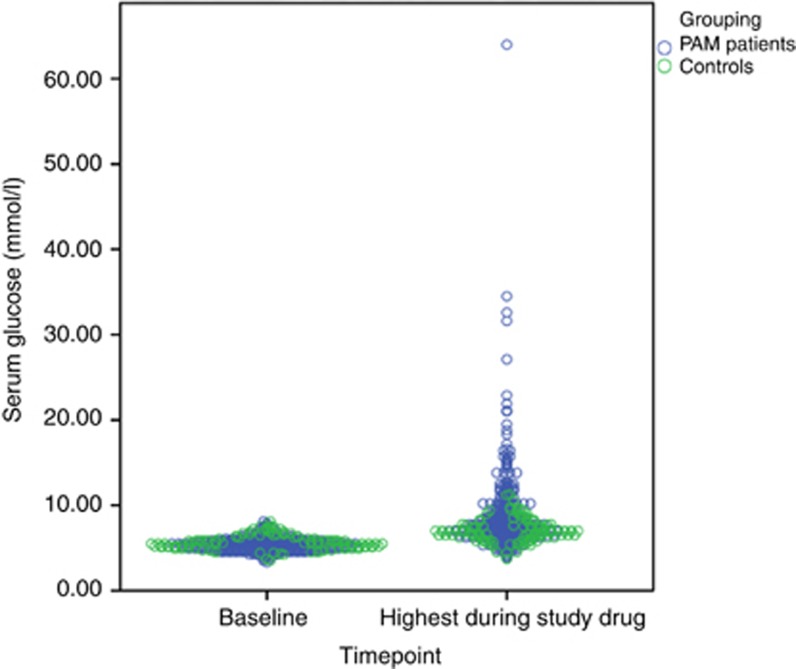
Baseline serum glucose levels and highest serum glucose levels during study drug exposure according to group.

The mean fasting glucose value at baseline (before receiving the first dose of the study drug in each trial) was within the normal range (upper limit of normal 6 mmol l^−1^) but significantly lower in the PAMi group compared with the control group, 5.4 mmol l^−1^ (95% CI 5.3–5.5) *vs* 5.7 mmol l^−1^ (95% CI 5.5–5.8; *P*=0.001). The mean highest blood glucose value during exposure to study drugs was significantly higher in the PAMi group 8.6 mmol l^−1^ (95% CI 8.2–9.1) *vs* 7.1 mmol l^−1^ (95% CI 6.8–7.4) in the control group, *P*=0.001 (Figure 1). However, there was no significant difference in the incidence of all-grade hyperglycaemia between cases (86.6%) and controls (80.7% OR: 1.54, 95% CI: 0.88–2.69, *P*=0.129). Importantly, a significantly higher number of patients treated with PAMi developed grade 3–4 hyperglycaemia compared with the control group, 26/387 (6.7%) *vs* 0/109 (0%), *P*=0.129 (Table 3).

Although the incidence of all-grade and high-grade hyperglycaemia was 86.6% and 6.7% in the case group, respectively, only a minority of patients (26/387 patients, 7%) needed treatment for persistent grade 3–4 hyperglycaemia after dosing with PAMi. Metformin (500–1000 mg daily) was sufficient to achieve good glycemic control in the majority of cases (22 patients, 5.7%). Only 4 patients (1%) required higher doses of metformin (up to 3000 mg per day). Metformin was usually prescribed according to the PAMi treatment schedule, for example, continuously with the continuous schedule and on dosing days in the intermittent schedule trials, achieving an effective glycemic control within few days. Only two patients (0.5%) required addition of a second oral hypoglycaemia agent and four (1%) required short-acting insulin. The study drug had to be withheld in nine patients (2.3% of the cases) due to high-grade hyperglycaemia. Also hyperglycaemia was reported as a dose-limiting toxicity (DLT) in 5 patients (1.3%) as per protocol guidelines.

The majority (63%) of patients in the case group developed hyperglycaemia during the first cycle of treatment. The highest hyperglycemic levels in 75% of patients were detected during the first cycle of treatment with PAM pathway inhibitors either as single agents or combination with chemotherapy or other targeted agents. Only 25% of patients presented with hyperglycaemia beyond cycle two of treatment.

Of note, preliminary analysis of the two trials (Molife *et al*, 2014; Roda *et al*, 2014) that included combinations with taxanes or platinum did not show major effect of the required standard steroid premedication on the overall toxicity profile.

### Complications of hyperglycaemia

Although hyperglycaemia is well described with some PAMi, it is not known if prolonged use of these agents can be associated with complications such as diabetic ketoacidosis. In this study group, following treatment with PAMi, only four patients (1%) had evidence of glycosuria and three patients (0.8%) of ketonuria. More importantly, ketonuria was not combined with metabolic acidosis or hypotension (classically associated with diabetic ketoacidosis), nor was it associated with abnormal serum electrolytes. Also, no patients developed symptoms of a hyperosmolar hyperglycemic nonketotic coma. No patients had to be admitted in hospital due to complications of hyperglycaemia.

### Comparing different classes of PAM inhibitors

We also investigated if hyperglycaemia could be more prevalent with a specific kinase inhibitor of the PAM pathway. For this analysis controls were excluded. The proportion of patients developing grade 3–4 was significantly different between different PAMi *P*<0.001 (Table 4). Therefore, multivariable logistic regression models were used to study the association between different types of PAMi and incidence of high-grade hyperglycaemia after correcting for potential covariates in our data set. Results are summarised in Table 5 and demonstrate that patients on AKT and multikinase inhibitors are at higher risk of developing grade 3–4 hyperglycaemia.

## Discussion and Conclusions

Agents inhibiting PI3K–AKT–mTOR pathway are currently at different stages of clinical development, with some already approved for advanced cancers. Metabolic complications associated with these agents, including hyperglycaemia and hyperlipidemia, are usually considered as on-target toxicities (Busaidy *et al*, 2012). Hyperglycaemia has been largely described with first-generation selective allosteric mTORi, everolimus and temsirolimus (Hudes *et al*, 2007; Motzer *et al*, 2008; Sivendran *et al*, 2014; Verges *et al*, 2014). However, we sought to determine the risk and complications of hyperglycaemia with a second generation of PAMi. In comparison with first-generation mTORi, PIK3*α* subunit-specific inhibitors, such as BYL719, are associated with a higher risk of hyperglycaemia described in literature as frequent as 49% of cases, particularly with higher doses. Although very frequent, in our experience hyperglycaemia is usually reversible with oral antihyperglycemic therapy or sometimes with short-term drug interruption (Gonzalez-Angulo *et al*, 2013). Drugs targeting all isoforms of PI3K (pan-PI3Ki) such as GDC-0941 (Garcia *et al*, 2011), BKM120 (Rodon *et al*, 2014) and CH5132799 (Blagden *et al*, 2014) are associated with varying degrees of hyperglycaemia, ranging from <10% in patients treated with GDC0941 to >30% with BKM120 (8% of high-grade). Hyperglycaemia with some pan-PI3Ki such as CH5132799 is dose dependent (Blagden *et al*, 2014). Conversely, other pan-PI3Ki, such as SAR245408 (Shapiro *et al*, 2014) and PX-866 (Hong *et al*, 2012), are not associated with a significant increase in blood glucose level.

Data regarding the risk of hyperglycaemia with AKTi are still at preliminary stages and again indicates the variability between different drugs. For example, the allosteric AKTi MK2206 has been associated with low-grade and transient hyperglycaemia (Yap *et al*, 2011; Molife *et al*, 2014). However, hyperglycaemia was more frequent with AKT kinase inhibitors such as AZD5363 (Banerji *et al*, 2013) and GDC-0068. The high incidence of hyperglycaemia in our data set is consistent with these findings. Furthermore another AKTi, the GSK690693 (Crouthamel *et al*, 2009), was significantly associated with hyperglycaemia in animal models and this limited its further clinical development.

Published or presented data of mTORC1/2 inhibitors such as AZD2014 (Banerji *et al*, 2012), INK-128 (Infante *et al*, 2012; Tabernero *et al*, 2012) and DS-3078a (Capelan *et al*, 2013) suggest that incidence of hyperglycaemia is not much different from first-generation mTORi. Data about INK-128 (Infante *et al*, 2012; Tabernero *et al*, 2012), comparable with our data set, reported hyperglycaemia as a frequent toxicity with an incidence of 44% for all-grade and 4% for high-grade with intermittent schedule. Significantly higher was the hyperglycaemia with the continuous dose schedule (88% for all-grade and 16% for high-grade). The mTORC1/2i AZD2014 (Banerji *et al*, 2012), has shown a comparatively lower incidence of hyperglycaemia (9%) while the incidence of all-grade hyperglycaemia for DS-3078a (Capelan *et al*, 2013) was 17%.

In this retrospective case-control study, we report that inhibition of different nodes in the PAM pathway is associated with significantly increased risk of high-grade hyperglycaemia (reported in 7% of the patients), compared with the control group treated with agents not directly targeting this pathway. All hyperglycemic events including high-grade events have always been clinically completely asymptomatic and transient. Importantly, high-grade hyperglycaemia was not associated with severe metabolic complications (no patients developed diabetic ketoacidosis or hyperosmolar hyperglycemic nonketotic state or showed marked electrolyte alterations). Treatment with usual therapeutic doses of metformin on dosing days of PAMi was sufficient to successfully treat hyperglycaemia.

A limited number of other factors predisposing to hyperglycaemia such as BMI and fasting glucose level were looked at and were not found to be significantly influencing grade 3–4 hyperglycaemia. It is important to note that inclusion criteria in phase I studies of PAMi excluded diabetics and we chose a cohort of non-diabetic controls and there is potential to significantly higher grade 3–4 hyperglycaemia, if diabetics were included. Interestingly hyperglycaemia was observed more significantly with multikinase inhibitors and AKTi but not mTORi when compared to PI3Ki. The biological reasons for these differences are not yet completely known. This information will need to be confirmed in larger cohorts, however, it is important information that can be used in the choice of which PAMi to use as single agent or in combination studies. Although a well-described on-target toxicity of PAMi, hyperglycaemia was reported, in our study, as a DLT in a minority of patients (5/387, 1.3%) leading to drug discontinuation in only 9 patients (2.3%).

In conclusion, our data confirm that hyperglycaemia with PAM pathway inhibitors is a common and manageable toxicity in non-diabetic cancer patients. There are subtle differences in the incidence of hyperglycaemia depending on the specific targets within the PAM pathway. Hyperglycaemia is an important but not a limiting factor to the further development of these drugs as single agents or in combination therapies.

## Figures and Tables

**Table 1:** Phase I clinical trials in cases and controls

**Cases**			**Controls**		
**Target**	**No. of trials**	**No. of patients**	**Target**	**No. of trials**	**No. of patients**
PI3K	4	78 (20.1%)	c-MET	1	20 (18.3%)
AKT	8	144 (37.2%)	HSP90	1	10 (9.2%)
mTORC	5	138 (35.6%)	MEK	1	12 (11%)
Multikinase PI3K/mTORC	1	27 (6.9%)	VEGF	2	26 (23.8%)
			EGFR	1	23 (21.1%)
			HDAC	2	13 (11.9%)
			INTEGRIN	1	4 (3.6%)
			IGF-1R	1	1 (0.9%)
Total	18	387	Total	10	109

Case group: 18 phase I clinical trials of PI3K–AKT–mTOR inhibitors single agents and in combination with chemotherapy or other targeted therapies.

Control group: 10 phase I clinical trials with agents not known to predominantly inhibit PI3K–AKT–mTOR pathway.

**Table 2:** Baseline patient clinical characteristics

	**Cases**	**Controls**
*N*	387	109
**Sex**		
Female	193 (49.9%)	53 (48.6%)
Male	194 (50.1%)	56 (51.4%)
Age, year median (range)	59.7 (22.25–81.08)	56.3 (17.49–88.3)
BMI, kg m^−2^ median (range)	25.93 (15–43)	27.33 (15–40)
**Performance status**		
0	106 (27.4%)	25 (22.9%)
1	277 (71.6%)	82 (75.3%)
2	4 (1.0%)	2 (1.8%)
Number of previous lines of chemotherapy	2 (0–11)	2 (0–11)
**Tumour type**		
Lung and mesothelioma	48 (12.4%)	11 (10.1%)
Colorectal	99 (25.6%)	16 (14.7%)
Gynaecological (ovarian, cervical, endometrial)	59 (15.2%)	6 (5.5%)
Breast	33 (8.5%)	15 (13.8%)
Prostate	22 (5.7%)	10 (9.2%)
RCC	17 (4.4%)	3 (2.8%)
Others	109 (28.2%)	48 (44.0%)
**RMH score**^a^		
0	85 (22.0%)	25 (22.9%)
1	129 (33.3%)	32 (29.4%)
2	107 (27.6%)	32 (29.4%)
3	52 (13.5%)	20 (18.3%)
Unknown	14 (3.6%)	–
Time on trial (days)	94.61 (1–1524)	124,75 (1–1096)

Abbreviations: BMI=body mass index; RMH=The Royal Marsden Hospital.

Baseline characteristics such as age at the time of recruitment, tumour type, number of previous lines of chemotherapy and performance status were balanced between cases and controls.

a RMH score: albumin+number metastatic sites+LDH.

**Table 3:** Incidence of all grades and high-grade hyperglycaemia in cases (PAM inhibitors) and controls (non-PAM inhibitors)

	**All grades hyperglycaemia**				**Grade 3 and 4 hyperglycaemia**		
	**Total**	**No**	**Yes**	***P*****-value**^a^	**No**	**Yes**	***P*****-value**^a^
Cases *n* (%)	387 (100)	52 (13.4)	335 (86.6)	0.129	361 (93.3)	26 (6.7)	0.005
95% CI	–	10.4%–17.2%	82.8%–89.6%		90.3%–95.4%	4.6%–9.7%	
Controls *n* (%)	109 (100)	21 (19.3)	88 (80.7)		109 (100)	0 (0)	
95% CI		13.0%–27.7%	72.3%–87.0%		96.6%–100%	0%–3.4%	

Abbreviation: CI=confidence interval.

a *χ*^2^-test.

**Table 4:** Incidence of hyperglycaemia between different inhibitors of the PI3K/Akt/mTOR (PAM) pathway

	**All-grade hyperglycaemia**				**High-grade hyperglycaemia**		
**Incidence**	**Total**	**No**	**Yes**	***P*****-value**^a^	**No**	**Yes**	***P*****-value**^a^
PI3K inhibitors, *N* (%)	78 (100)	16 (20.5)	62 (79.5)	0.053	77 (98.7)	1 (1.3)	<0.001
95% CI		13.0%–30.8%	69.2%–87.0%			0.2%–7.0%	
mTORC 1 or 2 inhibitors, *N* (%)	138 (100)	18 (13.0)	120 (86.7)		135 (97.8)	3 (2.2)	
95% CI		8.4%–19.7%	80.3%–91.6%		93.8%–99.3%	0.7%–6.2%	
AKT inhibitors, *N* (%)	144 (100)	18 (12.5)	126 (87.5)		128 (88.9)	16 (11.1)	
95% CI		8.1%–18.9%	81.1%–91.9%		82.7%–93.0%	7.0%–17.3%	
Multikinase inhibitors, *N* (%)	27 (100)	0 (0)	27 (100)		21 (77.8)	6 (22.6)	
95% CI		0%–12.5%	87.5%–100%		59.2%–89.4%	10.6%–40.8%	

Abbreviation: CI=confidence interval.

a *χ*^2^-test.

**Table 5:** Multivariate logistic regression analysis of factors associated with increased risk of grade 3–4 hyperglycaemia in patients receiving PAM inhibitors

**Variable**	**OR**	**95% CI**	***P*****-value**
Type of PAM inhibitor		–	0.002
PI3K inhibitors	1.0	–	–
TORC 1/2 inhibitors	1.08	0.01–12.25	0.950
AKT inhibitors	9.27	1.19–72.46	0.034
Multikinase inhibitors	17.57	1.94–158.80	0.011
Gender (female *vs* male)	0.74	0.28–1.98	0.545
Age (continuous variable)	0.960	0.92–0.100	0.037
BMI (continuous variable)	0.999	0.98–1.02	0.899
Hypertension (yes *vs* not)	1.528	0.32–7.36	0.597
Fasting glucose at baseline (continuous variable)	1.148	0.60–2.20	0.679

Abbreviations: CI=confidence interval; OR=odds ratio; PAM=PI3K–AKT–mTOR.

In ‘Type of PAM inhibitor', PI3K inhibitors are used as the reference group.
