# Supplementary material for: Complications of hyperglycaemia with PI3K–AKT–mTOR inhibitors in patients with advanced solid tumours on Phase I clinical trials
Source: Br J Cancer. 2015 Nov 10;113(11):1541–7. doi: 10.1038/bjc.2015.373 (PMC4705886; doi:10.1038/bjc.2015.373)
Supplement: Supplementary Information [file bjc2015373x1.docx]

|  | **SUPPLEMENTARY MATERIALS** |  |
| --- | --- | --- |


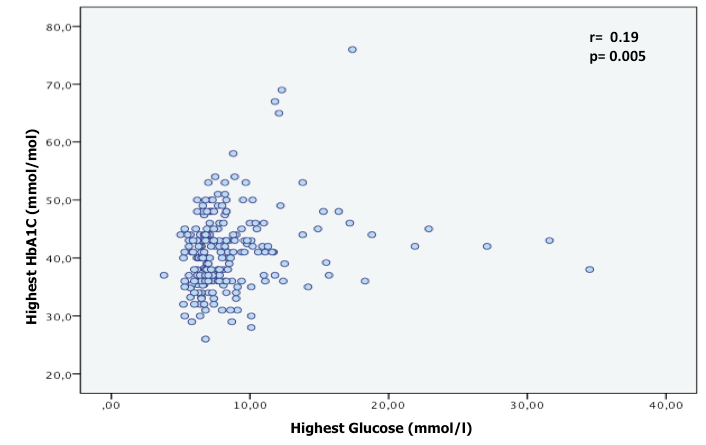


Supplementary Fig 1.

Correlation between glucose and HbA1c.


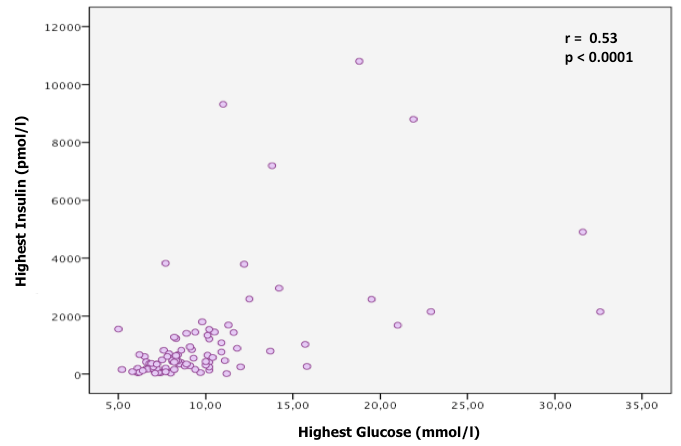


Supplementary Fig 2.

Correlation between glucose and insulin.


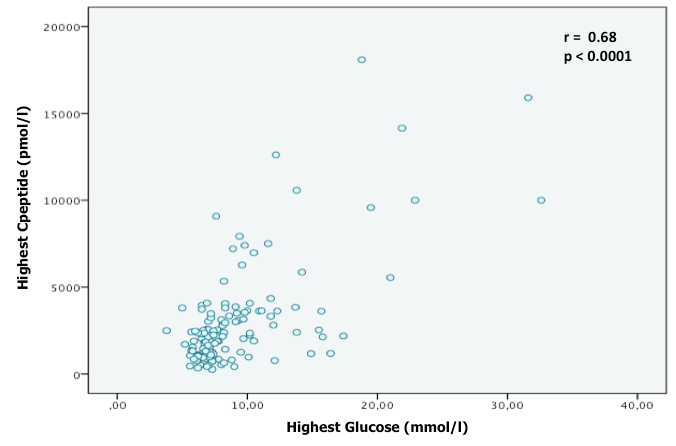


Supplementary Fig 3.

Correlation between glucose and c-peptide.
